# The Macro- and Micro-Mechanics of the Colon and Rectum II: Theoretical and Computational Methods

**DOI:** 10.3390/bioengineering7040152

**Published:** 2020-11-25

**Authors:** Yunmei Zhao, Saeed Siri, Bin Feng, David M. Pierce

**Affiliations:** 1 Department of Biomedical Engineering, University of Connecticut, Storrs, CT 06269, USA; yunmei.zhao@uconn.edu (Y.Z.); siri@uconn.edu (S.S.); fengb@uconn.edu (B.F.); 2 Department of Mechanical Engineering, University of Connecticut, Storrs, CT 06269, USA

**Keywords:** colorectum, biomechanics, afferent endings, mechanotransduction, constitutive modeling, theoretical and computational models

## Abstract

Abnormal colorectal biomechanics and mechanotransduction associate with an array of gastrointestinal diseases, including inflammatory bowel disease, irritable bowel syndrome, diverticula disease, anorectal disorders, ileus, and chronic constipation. Visceral pain, principally evoked from mechanical distension, has a unique biomechanical component that plays a critical role in mechanotransduction, the process of encoding mechanical stimuli to the colorectum by sensory afferents. To fully understand the underlying mechanisms of visceral mechanical neural encoding demands focused attention on the macro- and micro-mechanics of colon tissue. Motivated by biomechanical experiments on the colon and rectum, increasing efforts focus on developing constitutive frameworks to interpret and predict the anisotropic and nonlinear biomechanical behaviors of the multilayered colorectum. We will review the current literature on computational modeling of the colon and rectum as well as the mechanical neural encoding by stretch sensitive afferent endings, and then highlight our recent advances in these areas. Current models provide insight into organ- and tissue-level biomechanics as well as the stretch-sensitive afferent endings of colorectal tissues yet an important challenge in modeling theory remains. The research community has not connected the biomechanical models to those of mechanosensitive nerve endings to create a cohesive multiscale framework for predicting mechanotransduction from organ-level biomechanics.

## 1. Introduction

The macro- and micro-scale biomechanics of the colon and rectum (colorectum) play critical roles in many pathophysiological conditions in the lower gastrointestinal tract (GI), and thus have attracted growing research attention focusing on experimentally characterizing colorectal biomechanics in both health and disease. Abnormal colorectal biomechanics and/or mechanotransduction associate with an array of GI diseases, including the inflammatory bowel disease [1], irritable bowel syndrome [2], diverticula disease [3], anorectal disorders [4], ileus [5], and chronic constipation [6]. Common to all those disease conditions are biomechanical factors working on the nervous system of the large intestine to collectively affect the motilities of the gut and/or mechanotransduction.

The colorectum is a tubular structure consisting of two major composite layers loosely connected at an interstitial space between the submucosa and the circular muscular layers [7]. The inner composite constitutes submucosal and muscular layers, while the outer composite includes the circular muscular, longitudinal muscular, and serosal layers. We summarized the layered structure of the large intestine, and the respective functional roles of these layers in both health and disease, in our companion review [8]. Prior multi-scale experimental studies informed our understanding of colorectal biomechanics, including results from histological investigations [7,9,10] and mechanical tests [7,9,10,11,12,13,14,15,16]. We summarize this knowledge about colorectal biomechanics in the following: (1) longitudinal mechanical heterogeneity—the biomechanical properties and concentrations of collagen vary from colonic, intermediate, and rectal locations; (2) through-thickness mechanical heterogeneity—the internal composite of the mucosa and submucosa differs from the outer composite of two muscular layers and the serosa; (3) microstructural heterogeneity—collagen fibers concentrate in the submucosa and to a lesser extent in the serosa; and (4) longitudinal heterogeneity in the residual stresses [7,10,12,15].

All layers of the colorectum also present extrinsic and intrinsic nerve endings except the serosa [17,18]. These fine nerve endings, microns in diameter, are embedded in the fine structures of colorectal tissues and directly interact with local mechanical stresses and strains around individual endings. More than 70% of the extrinsic sensory nerve endings in the colorectum are mechanosensitive [17], i.e., capable of encoding local mechanical stresses or strains into trains of action potentials informing the central nervous system. Prior experimental studies provided the following observations on the neuroanatomy and neurophysiology of the colorectum. (1) The colorectum is innervated by the extrinsic sensory afferents whose somata are in the thoracolumbar and lumbosacral dorsal root ganglia. They send sensory endings to the colorectum via the lumbar splanchnic and pelvic nerves, respectively. (2) The lumbar splanchnic afferents encode noxious mechanical probing of the colorectum while the pelvic afferents encode both noxious mechanical probing and physiological levels of colorectal distension and luminal shearing. (3) Most, if not all, colorectal afferents are unmyelinated C-fibers with small axonal diameter (∼1 micron) and slow conduction velocity (<1 m/s for mouse afferents). (4) Afferent nerve density is highest in the myenteric plexus between the two muscular layers, as well as in the submucosa right below the mucosa. (5) Intrinsic neurons residing within, i.e., the enteric nervous system (ENS), also innervate the colorectum, and consist of the submucosa plexus and myenteric plexus. Myenteric plexus is mainly responsible for coordinated intestinal motility to transmit luminal contents down the GI tract, while submucosal plexus controls the gland secretion into the GI lumen.

To better understand the underlying mechanical mechanisms of visceral pain, and establish potential treatment targets, the development of robust computational models is crucial to predicting organ/tissue biomechanics and their connection to sensory encoding of mechanical forces. A key requirement for such a computational model is the development of suitable constitutive relations that characterize the tissue mechanics, i.e., a mathematical formulation for the stress-strain relation. The assumed constitutive relations must then be fit to experimental data under loading conditions of interest and can be validated by successfully predicting results from independent experiments.

Here we aim to review the theoretical and computational models currently established for predicting the biomechanics and mechanobiology of the colorectum. We will first review the current literature on computational modeling of colon and colorectum biomechanics and of stretch-sensitive afferent nerve endings. We will then highlight our recent advances in modeling both organ and intra-tissue biomechanics in the colorectum, and of stretch-sensitive afferent endings. While current models provide insight into the organ-level biomechanics and the stretch-sensitive afferent nerve endings of colorectum tissues independently, an important challenge in modeling theory remains. We, as a community, have yet to connect the biomechanical models to models of mechanosenstive nerve endings to create a cohesive multiscale framework for predicting mechanotransduction from organ-level biomechanics.

## 2. Modeling the Colorectum: State of the Art

### 2.1. Macro-Scale: Biomechanics of the Colorectum

The colon undergoes large deformations in vivo, distending up to 30% radially to accommodate variable quantities of fecal matter [15], and thus mechanical analyses of the colon should employ large-strain kinematics and nonlinear mechanics. The framework of hyperelasticity is generally employed to handle the large-strain kinematics, i.e., geometric nonlinearities. There are two main categories of constitutive models proposed for the colorectum: purely phenomenological models using mathematical functions chosen to provide best fits to experimental data (without regard to microstructure), and structure-based models using mathematical functions and/or parameters motivated by the underlying microstructure, cf. [19,20]. The development of constitutive models for other tubular soft tissues, i.e., arteries [21,22,23,24], duodenum [25], and esophagus [26,27], has significantly influenced constitutive models proposed for the colon and rectum. As summarized in Table 1, we review the state-of-the-art in constitutive models proposed for the colorectum and the various experimental data used to fit these models, including both the mechanical test and the species of the donor.

Researchers implemented several phenomenological constitutive models to simulate colorectal biomechanics by fitting with experimental data. Higa et al. [28] coupled the Mooney-Rivlin hyperelastic model with a convolution integral to capture the viscoelastic mechanical response of the bulk composite colon from goat. They validated their model using compression tests in-vivo, a mechanical test with relevance to modeling surgical procedures. Additionally, the model is isotropic while the microstructure of colon includes muscle fibers and networked collagen that generate mechanical anisotropy. Bellini et al. [11] proposed a phenomenological constitutive model for three distinct portions of the porcine small intestine (ileum, jejunum, and duodenum). They performed planar biaxial extension tests on ileum, jejunum, and duodenum, and fitted these data to determine regional model parameters for the orthotropic Fung-type exponential model. Their four-parameter model captured the longitudinal heterogeneity of porcine small intestine as they validated each fitting through extensive numerical simulations. Unfortunately the Fung model cannot directly capture the degree of anisotropy (resulting from the underlying microstructures) so the authors evaluated the stiffnesses in two orthogonal directions, i.e., axial and circumferential. Furthermore, limited by the experiments, the work focused on the bulk composite and neglected the mechanics of the distinct tissue layers within the colorectal wall. Sokolis et al. [12] also implemented the Fung-type exponential formulation with four parameters to fit experimental data from inflation-extension tests at different regions of rat large intestine (at proximal, transverse, distal colon and rectum). This study reports a first approach in modeling the physiologically-relevant multiaxial behavior of passive large intestine. Similar to Bellini et al. [11], the assumption of through-thickness homogeneity is the primary limitation.

Sokolis [29] fitted three phenomenological constitutive models to pressure-diameter data from inflation-extension tests of eight different regions of small intestine from rats: the four-parameter Fung, the seven-parameter Chuong and Fung, and the seven-parameter Tong and Fung models. The models provide reasonable fits to the data but suffer from the limitations noted above.

These phenomenological models addressed the nonlinear, hyperelastic responses of bulk colon, and characterized the longitudinal biomechanical heterogeneity of the colon. However, the models above all assumed the colon was transmurally homogeneous and thus fail to characterize the layered structure of the colon wall, as well as the various microstructures within these layers. Such shortcomings also mean that the phenomenological models cannot predict the intra-tissue distributions of local stresses [30].

Beyond these phenomenological models, structure-based constitutive models, guided by microstructural data from large intestine usually acquired via imaging methods, appear in the literature on colorectal biomechanics. This structure-based approach allows these models have a clear correlation between model parameters and microstructural features of the large intestine. Specifically, these constitutive models (strain-energy functions) account for the contributions of different constituents of the colon, e.g., an isotropic ground matrix and embedded fibers (collagen and/or muscle). Ciarletta et al. [31] decoupled the strain-energy density into an isotropic ground matrix and anistropic contributions from four families of directional fibers They fit this model to both uniaxial extension and shear tests of pig colon. This model successfully reproduced the passive mechanical response of the tissue by leveraging the preferred directions of fiber reinforcements. Although the model involved the fiber-reinforcement present in the layered tissue, it still focused on the bulk composite and failed to address the layer-specific properties. Guided by inflation-extension tests and observations via optical microscopy, Sokolis and Sassani [13] established a constitutive model which includes a neo-Hookean ground matrix and five families of fibers by fitting with experimental data at four regions (ascending, transverse, and descending colon, and rectum) of rat colons. Compared to the previous work, (cf. [12]), the proposed ten-parameter model directly reflects the underlying microstructure, and the tests reflect a physiologically-relevant range of deformations, to increase the fidelity of the model. While the model accounted for contributions from multiple families of fibers, it does not consider through-thickness heterogeneity. On the basis of multiple uniaxial tensile tests of pig colon, two studies (Ciarletta et al. [31], Carniel et al. [9]) established fiber-reinforced, hyperelastic constitutive formulations to capture families from both collagen and muscle fibers. Carniel et al. [9] selected a three-fiber-family model to describe the fibrous microstructure in the layered tissue (two families in the submucosa and one family in the muscular layers), to fit data generated from uniaxial extension tests from six orientations of loading. Again, this model addressed the different fiber families very well, but fails to capture through-thickness heterogeneity resulting from the layered structure. Additionally, preparation of the specimens may also damage the connectivity of fiber components and thus reduce the fidelity of the resulting model. Patel et al. [15] proposed a structure-based constitutive model for two regions of the colon (spiral and descending), and accounted for residual strains, based on the passive mechanical behavior of the swine colon. Their model includes a neo-Hookean ground matrix with embedded families of longitudinal and circumferential muscle fibers, and two families of dispersed collagen fibers. They validated the predictive power of their model against inflation-extension tests at physiologically relevant pressures.

Very few of the structure-based models for colonic and rectal tissues are based on biaxial extension tests. Puértolas et al. [16] tested the ability of five structure-based constitutive models to fit data from biaxial extension tests carried out on different segments of colons from pigs. They tested constitutive models including both discrete-fibers (with two [23] or four [24] families, and two dispersed [32] families) and orientation distribution functions (ODFs, the microfiber von Mises model [33] and microfiber Bingham model [34]) to describe the fibrous microstructure of the colon. The constitutive model with four families of fibers (and eight parameters) demonstrated the most predictive power. The two models with ODFs showed comparable accuracy in predicting data as the model with two families of dispersed fibers (all using five parameters). In contrast, the model with two families of embedded fibers using four parameters showed the worst predictive power. These models will likely predict organ-level stresses, strains, and deformations. In the absence of formulations for individual colorectal layers, these models cannot predict intra-tissue distributions in stresses and strains, particularly through the thickness.

Recently, we proposed an ODF-based constitutive model for colorectal tissue regions (colon, intermediate and rectal) where we specified the ODF via a symmetric, positive-definite diffusion tensor [35] which determines the local 3-D orientation distribution of the fiber network in individual layers. We fit the model to reliably reproduce results from biaxial extension tests on colon segments from mice [7,10]. We also implemented the constitutive model in a finite element framework, accounted for experimentally determined residual stretches/stresses, and successfully predicted independent biaxial extension and pressure-diameter experiments.

These state-of-the-art constitutive models can predict a comprehensive range of mechanical responses of the colon tissue on the basis of the coupled experimental-computational approach. Establishing reliable structure-based constitutive models strongly benefits from experimental measurements of the microstructural parameters, such as the distribution of collagen and muscle fibers. The same experimental data can inform fitting of any of the models detailed above thus facilitating direct comparison of constitutive models, cf. [16]. Additionally, there is generally a trade-off based on the number of model parameters, i.e., the complexity of the model. The ease of fitting and using the model favors models with fewer parameters, whereas the predictive power of the model (to better fit experimental results) is generally enhanced with increasing complexity of the models.

### 2.2. Micro-Scale: Mechanotransduction of the Colorectum

Studies on mechanotransduction generally rely on computational simulations because the experimental approaches currently available can not directly measure the micromechanical stresses and strains around individual nerve endings in the colorectum. The generation of action potentials in mechanotransduction is usually simulated by neural membrane models, which consider the lipid bilayer membrane and transmembrane ionic currents as a capacitance in parallel with conductance and potential sources. The theoretic framework of the neural membrane model was originally developed by Nobel Prize laureates Hodgkin and Huxley in 1950s [36] and was frequently adopted or modified by recent models, including the Markov-typed formulation [37,38]. Models generally divide the three-dimensional structure of neuronal tissues into small isopotential compartments, each simulated by a neural membrane model. Spatial discretization allows researchers to derive numerical solutions of intracellular and extracellular membrane voltages in both spatial and time domains. Transmembrane potentials directly reveal the action-potential events during mechanotransduction. Mechanosensitive ion channels, whose conductance is a function of membrane mechanical stress or strain, are the key driver of changes in transmembrane potential.

Only a few candidate mechanosensitive ion channels have been discovered which has prevented theoretical studies on mechanostransduction [39]. Piezo2 channels, for example, seem to dominate the mechanostransduction of slow-adapting Merkel endings innervating the skin [40], and this understanding facilitated a computational model simulating the mechanotransduction of Merkel endings innervating the skin [41]. In contrast, the molecular identities of mechanosenstive ion channels remain poorly understood for mechanosensitive nerve endings in the colon and rectum, which are mostly free nerve endings without myelination. We pioneered the first theoretical model that simulated the mechanotransduction of afferent endings in the colorectum [42]. Our model incorporated a novel mechanosenstive ion channel that drives the generation of action potentials. Through a simple lumped parametric model, we successfully derived microscale membrane tension at the nerve endings driven by macroscale colorectal distension. By incorporating independent Markov models that recapitulated subtypes of sodium channels, the model provided new evidence to suggest important roles of ${\mathrm{Na}}_{V}1.6$ in encoding tonic spiking by stretch-sensitive sensory nerve endings.

## 3. Towards Coupled Multi-Scale Modeling of the Colorectum

Here we review our recent efforts towards establishing a coupled multi-scale model of the colorectum.

### 3.1. Constitutive Models of the Biomechanics

We recently established and validated a constitutive model for mouse colorectum capturing longitudinal and through-thickness biomechanical heterogeneity [43]. Briefly, we modeled the individual mechanical responses of the inner and outer composites using an additive decomposition of the isochoric strain energy

(1) $$\bar{\Psi }={\bar{\Psi }}_{\mathrm{IM}}+{\bar{\Psi }}_{\mathrm{FN}},$$

with a contribution from an isotropic neo-Hookean matrix ${\bar{\Psi }}_{\mathrm{IM}}$ where μ>0 is the shear modulus of the underlying matrix, and with a contribution from a network of fibers [35,44]

(2) $${\bar{\Psi }}_{\mathrm{FN}}=\int_{\Omega }\rho \left(M\right)\frac{k_{1}}{2k_{2}}\left(\exp\left[k_{2}{\left({\bar{I}}_{4}-1\right)}^{2}\right]-1\right)H\left({\bar{I}}_{4}-1\right)d\Omega ,$$

where $k_{1}>0$ is a stress-like material parameter, $k_{2}>0$ is a dimensionless parameter, ${\bar{I}}_{4}=M\;\cdot \;\bar{C}M$ is the isochoric fourth pseudo-invariant of M (the reference angular orientation of a single fiber), and H is a Heaviside function evaluated at $\left({\bar{I}}_{4}-1\right)$, i.e., the collagen fibers only support tension. Here ρ(M) is an orientation distribution function (ODF) characterizing the local angular density of the fiber network as [35]

(3) $$\rho \left(M,D\right)=\frac{\sin\theta }{{\left|D\right|}^{1/2}{\left(M^{T}D^{-1}M\right)}^{3/2}}.$$

where D is a second-order, symmetric, positive-definite tensor and with $1/4\int_{\Omega }\rho \left(M\right)d\Omega =1$, where $\Omega =M\in R^{3}:\left|M\right|=1$ is the unit sphere. We specified ρ(M) using a second-order, symmetric, positive-definite tensor D

(4) $$D=\left[\begin{array}{ccc} D_{\theta \theta } & D_{\theta z} & D_{\theta r}\\ D_{\theta z} & D_{zz} & D_{zr}\\ D_{\theta r} & D_{zr} & D_{rr} \end{array}\right],$$

and particularized by defining α as the in-plane angle between the principal orientation of fibers and the circumferential direction such that $D_{\theta \theta }=\cos\alpha$, $D_{zz}=\sin\alpha$, and $D_{rr}=D_{\theta z}=D_{zr}=D_{\theta r}=0$ in (4), and where θ, *z*, and *r* are the local circumferential, longitudinal, and radial directions within the colorectum. We determined four model parameters (μ, $k_{1}$, $k_{2}$, α) from fitting our experiments in biaxial extension [7,10]. Specifically we determined the model parameters for individual layers of the colorectum using nonlinear optimization to fit our experimental stress-strain relations on layer-separated colonic, intermediate, and rectal segments.

### 3.2. Biomechanics: Computational Modeling and Results

**Figure 1:**
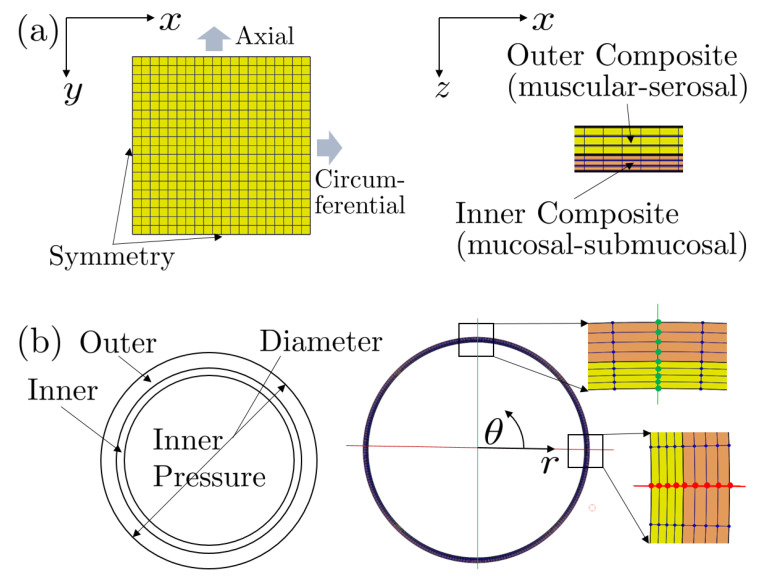
Finite element analyses for validation: (**a**) Biaxial extension test of the bulk coloretum via a two-layered, residually stressed specimens. The specimens have dimensions of 3.5 × 3.5 mm${}^{2}$ based on the symmetry boundary conditions, and biaxial load is applied through the linearly increased circumferential and longitudinal (axial) displacements. (**b**) Pressure-diameter test of the colorectum under applied intraluminal pressure via a two-layered, residually stressed tubular segments. Exploiting plane-strain conditions we modeled only 2 mm longitudinal segments of intact colorectums, and applied symmetry boundary conditions and fixed two radial rows of nodes normal to the radial direction (to prevent rigid-body rotations). We axially stretched the model by 30%, consistent with the experiments ex vivo, and the linearly increased intraluminal pressure from 0 to 100 mmHg [43].

To validate the predictive power of our constitutive models and modeling framework, we modeled the colorectum as a two-layered, residually stressed composite and established finite element (FE) models in FEBio (R2.8.5, University of Utah, Salt Lake City, UT, USA) [45] to reproduce experiments of (a) the biaxial extension tests of the reconstructed bulk composite and (b) the pressure-diameter test of the intact tubular composite, see Figure 1.

We incorporated the distribution of residual stretches/stresses using experimental measurements of circumferential residual stretches released by separating the layers, and implemented these via the prestrain algorithm build into FEBio [45,46]. Specifically, we assumed the stretches were homogeneous within each element and defined the prestretch by imposing a deformation gradient tensor

(5) $$F_{p}=\left[\begin{array}{ccc} \lambda_{p,r} & 0 & 0\\ 0 & \lambda_{p,\theta } & 0\\ 0 & 0 & \lambda_{p,z} \end{array}\right],$$

presented in cylindrical coordinates (θ, *z*, *r*), where $\lambda_{p,r}$, $\lambda_{p,\theta }$, and $\lambda_{p,z}$ are layer-specific, prescribed prestretches. We prescribed prestretches $\lambda_{p,\theta }$ for the inner and outer composites as determined experimentally, while the remaining prestretches $\lambda_{p,r}=\lambda_{p,z}=1$.

**Figure 2:**
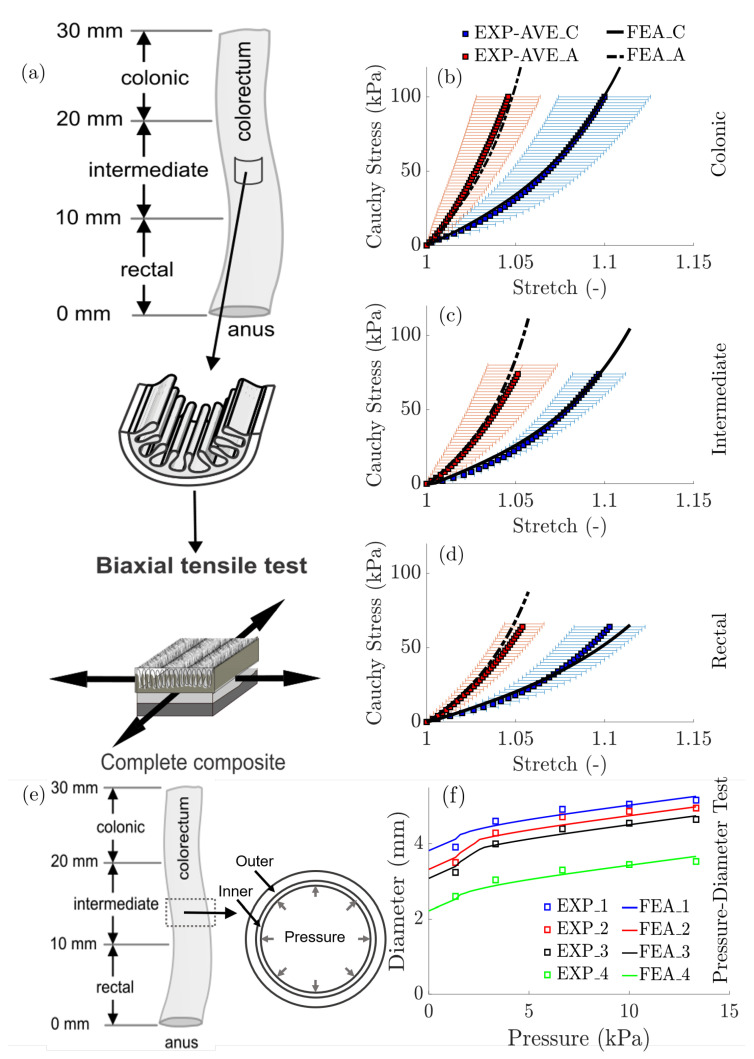
Simulation results: (**a**) Schematic diagram of the biaxial extension tests performed on three locations of bulk composite colorectum. (**b**–**d**) Mean experimental data (red and blue squares, plus error bars for standard deviation) with corresponding model predictions (solid and dashed curves) for (**b**) colonic, (**c**) intermediate, and (**d**) rectal bulk composite specimens of colorectum undergoing biaxial extensions. EXP = EXPeriment, AVE = Mean, FEA = Finite Element Analyses, C = Circumferential, and A = Axial. (**e**) Schematic diagram of the pressure-diameter tests performed on the tubular specimens of the coloretum. (**f**) Comparison of FE analyses with experimental measurements of diameter changes vs. increased pressure (adapted from [43]).

We validated our constitutive models and modeling framework by successfully predicting independent experimental data from both biaxial extension tests of bulk composite (intact) colorectal specimens and pressure diameter tests from tubular segments of colorectums, see Figure 2.

In Figure 2a–d, the FE predictions of the biaxial extension tests for the three longitudinal locations agree well with the averaged experimental data collected from an independent cohort of bulk (intact) specimens, indicating strong mechanical heterogeneity with decreased longitudinal and circumferential stiffness from the colonic to the rectal locations. In Figure 2e,f, the FE predictions of the pressure-diameter tests agree well with four independent experiments and there was no statistically significant difference in the pressure-diameter responses from segments of tubular colorectums from different longitudinal locations (colonic, intermediate, and rectal) despite the clear longitudinal heterogeneity in the biomechanical properties (model parameters). This consistent pressure-diameter relation along the longitudinal direction is likely from the combined effect of increased colorectal wall thickness and reduced mechanical stiffness from the colonic to rectal regions.

### 3.3. Theoretical Models of Stretch-Sensitive Afferent Endings

The nerve membrane contains three main types of ion channels, i.e., potassium (K) ions, sodium (Na) ions, and the leakages (L). If there is an imbalance in current across the membrane such that more positive charge enters the cell than leaves it, this naturally changes the membrane potential and causes it to depolarize (and vice versa). The membrane potential begins with the current flow through the membrane, where the total current $I_{m}$ is the sum of three ionic components and one capacitative (C) component from the Hodgkin-Huxley-like models [36],

(6) $$I_{m}=I_{K}+I_{\mathrm{Na}}+I_{L}+I_{C},$$

where the ionic currents $I_{K}$, $I_{\mathrm{Na}}$, and $I_{L}$ reflect the movement of potassium, sodium, and leakage ions through the membrane respectively.

We simplified the ionic currents from Na, K, and leak channels by Ohmic relations between channel conductances and the potential difference between the membrane potential and the reversal potential of that particular ion

(7) $$I_{\mathrm{ion}}=g_{\mathrm{ion}}\left(V_{m}-E_{\mathrm{rev}}\right),$$

where $I_{\mathrm{ion}}$ is the ionic current, $g_{\mathrm{ion}}$ is the conductance, $V_{m}$ is the membrane potential, and $E_{\mathrm{rev}}$ is the reversal potential. The potential difference ($V_{m}-E_{\mathrm{rev}}$) is the net driving force for ion flow. The conductance generally depends on the membrane voltage, and the maximum conductance (${\bar{g}}_{ion}$) occurs when all the channels are open.

The capacitive current $I_{C}$ correlates with the membrane voltage as

(8) $$I_{C}=C_{m}\frac{dV_{m}}{dt},$$

where $C_{m}$ is the membrane capacitance.

### 3.4. Mechanotransduction: Computational Models and Results on Stretch-Sensitive Afferent Endings

**Figure 3:**
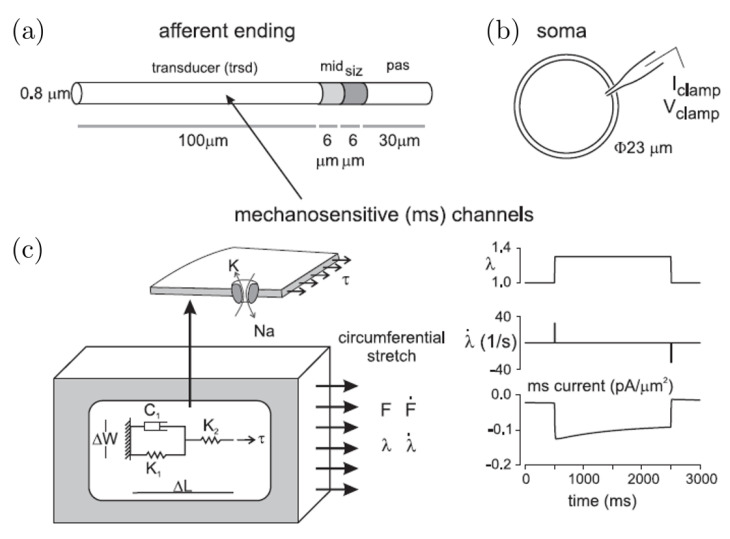
Schematic diagrams of our computational simulations of action potential generation in a mechanosensitive colorectal afferent endings. (**a**) We modeled the afferent ending as a four-segment cylinder consisting of a transducer zone (trsd), a spike initiation zone (siz), a transition zone in the middle (mid), and a passive conducting zone (pas). (**b**) We also included a representative mechanosensitive current in response to a stepped colorectal stretch at the afferent ending. (**c**) The afferent ending model simulates encoding of mechanical stretch by inclusion of mechanosensitive (ms) channels in the trsd zone gated by the membrane tension. We used a lumped parametric model to translate bulk colorectal deformation (force F, stretch ratio λ, and their first derivatives, $\dot{F}$, $\dot{\lambda }$) to the membrane tension at the afferent ending (adapted from [42]).

We simulated, using NEURON [47], the electrophysiological properties of colorectal afferent endings [42]. Briefly, we established a multi-compartment, cylindrical model to represent the fundamental morphological and electrical features of mouse colorectal afferent endings, see Figure 3a.

These computational simulations emulate AP (action potential) encoding, assuming a region of transducer terminal contiguous with a single spike-initiation zone (siz), similar to those used to simulate the axon initial segment (AIS) of neurons in the central nervous systems. Our model comprises a transducer zone (trsd), which produces a generator potential by depolarizing current from mechanosensitive channels (ms channels), a siz where the generator potential evokes AP spikes, and a middle section (mid) in which channel densities ${\mathrm{Na}}^{+}$ and $K^{+}$ gradually increase from the trsd side to siz side (to simulate the gradual change in ion channel densities). The passive compartment (pas) distal to siz provides space for axial diffusion of intracellular ${\mathrm{Na}}^{+}$ and $K^{+}$ ions. To achieve spatial and temporal accuracy in our simulations, we further divided the compartments into a total of 23 segments (10 in trsd, 5 each in siz and mid, and 3 in pas).

To recapitulate experimental findings, we incorporated four ${\mathrm{Na}}^{+}$ conductances and three $K^{+}$ conductances within the computational model, with independent Markov-type models (${\mathrm{Na}}_{V}1.6$ [48] and ${\mathrm{Na}}_{V}1.7$ [38]) and Hodgkin-Huxley-type models (${\mathrm{Na}}_{V}1.8$ [49], ${\mathrm{Na}}_{V}1.9$ [49]; $K_{A}$, $K_{S}$, and $K_{D}$ [50]).

**Figure 4:**
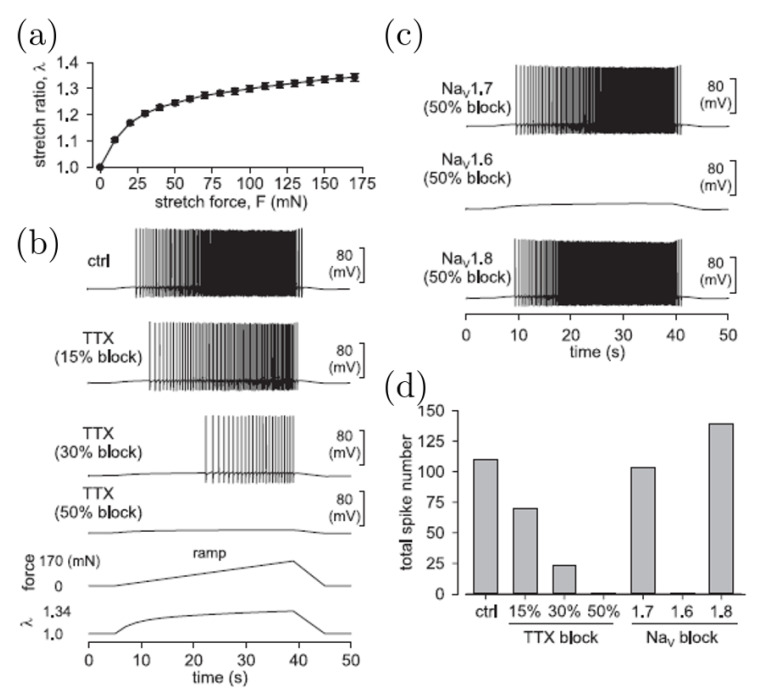
Simulations of localized serosal application of tetrodotoxin (TTX) and selective ${\mathrm{Na}}_{V}1.6$ and ${\mathrm{Na}}_{V}1.7$ blockers on stretch-sensitive colorectal afferent endings. (**a**) The ramped circumferential stretch λ determined experimentally. (**b**,**c**) We used the measured mean stretch to drive the afferent ending model for action potential generation: (**b**) TTX and (**c**) ${\mathrm{Na}}_{V}1.6$, ${\mathrm{Na}}_{V}1.7$, and ${\mathrm{Na}}_{V}1.8$. (**d**) The predicted total spike numbers (adapted from [42]).

In Figure 4 we show the simulated response of an afferent ending to a ramped colorectal stretch.

We recorded the ramped force, which drove the generation action potentials at the siz in the model, see Figure 3a, while ramping the circumferential stretch. We mimicked pharmacological blockage of ion channels with tetrodotoxin (TTX) by gradual reduction of the maximum conductances of both ${\mathrm{Na}}_{V}1.6$ and ${\mathrm{Na}}_{V}1.7$ in the model by 15%, 30%, and 50% separately, see Figure 4b. We simulated the pharmacological blockage of subtype-selective ${\mathrm{Na}}_{V}$ channels by reducing the corresponding maximal conductance by 50%, see Figure 4c. We summarized the total number of spikes evoked by the ramped stretch stimulus, and this suggested a key role for ${\mathrm{Na}}_{V}1.6$ in mechanotransduction by mechanosensitive nerve endings in the colorectum, see Figure 4d.

## 4. Discussion and Outlook

Future efforts should aim to improve the utility and reliability of existing frameworks for modeling the biomechanics and mechanobiology of the colorectum, which requires an integrated approach of combining imaging, mechanical testing, and theoretical and computational modeling. Computational approaches play an essential role in investigating the biomechanical behaviour of biological tissues and organs, and particularly aid in interpreting and understanding phenomena underlying experimental findings or predicting the impact of potential treatments for gastrointestinal diseases. Constitutive modeling in particular is essential in capturing the biomechanical heterogeneity of the colorectum for computational models. The fiber-reinforced constitutive formulations depend strongly on the parameters identified from experimental data, which demand (1) imaging approaches to exam the multi-layered structure and microstructure of the tissue, i.e., collagen and muscle fibers, and (2) mechanical tests for both bulk composite and individual layers, to probe strain-stress states closely mimicking the physiological conditions [16]. Models of the mechanosensitive sensory afferent endings allowed us to predict distinct mechanostransduction and biochemical pathways, such as stretch-activated ion channels, cell connectivity, and neuron signaling, and aid our understanding of stretch-activated sensory nerve endings. Since the mechanosensitive ion channels [39] convert mechanical stimuli into electrical membrane depolarization/repolarization in a biophysical process, it is also essential to develop a computational framework for modeling the neuron membrane.

While current models provide insight into the organ-level biomechanics and the mechanosensitive afferent endings of colorectal tissues independently, an important challenge in modeling theory remains. We, as a community, have yet to connect the biomechanical models to models of mechanosenstive nerve endings to create a cohesive mulitiscale framework for predicting mechaniotransduction from organ-level biomechanics. Generally the biomechanical analyses of organs and tissues involves finite element modeling that calculates intra-tissue distributions of strain and stress, and perhaps even electrical potentials generated during mechanical stimulation. We could then couple such information to biophysical, cellular models of neurons for simulating the propagation of electrical signals.

There are pioneering theoretical efforts coupling biomechanics and mechanotransduction, e.g., [41,51,52,53]. However, none of such research efforts studied mechanotransduction in visceral organs like the colon and rectum.

In prior research we did, via a custom-built stretch-sensitive ion channel, drive the initiation of action potentials with a coupled neural membrane model [42]. Nevertheless, our understanding of mechanotransduction in the colorectum will be enhanced by incorporating more realistic mechanical models, e.g., [43].

Development of such theoretical and computational methods for the distal colon and rectum will facilitate deeper understanding of the colorectum in health and disease, and may provide a platform for identifying, testing, and validating treatments for visceral pain and other ailments of the colorectum.

## Figures and Tables

**Table 1:** Constitutive models applied to model colon tissues including the reference, the constitutive model, the number of parameters, and the experimental model used for determining the parameters (mechanical test and donor species); where DEM = Demiray; dis fib = exponentially stiffening family of fibers with dispersed orientations about the principal direction; fiber = exponentially stiffening family of fibers; M-R = Mooney-Rivilin; N-H = neo-Hookean; ODF = orientation distribution function (with function specified thereafter); and visco = viscoelastic (time dependent) addition to the hyperelastic model. * Specifying the diffusion tensor generally requires six parameters, but with assumptions these reduced to a single parameter.

Reference	Model	Parameters	Experimental Data
Higa et al. (2007)	M-R + visco	4	in-vivo compression (goat colon)
Ciarletta et al. (2009)	N-H + 4 fiber	8	uniaxial extension + shear (pig colon)
Bellini et al. (2011)	Fung	4	biaxial extension (pig small intestine)
Sokolis et al. (2011)	Fung	4	inflation-extension (mouse intestine)
Sokolis + Sassani (2013)	N-H + 2 fiber	5	inflation-extension (rat intestine)
	N-H + 3 fiber	6	
	N-H + 4 fiber	8	
	N-H + 5 fiber	10	
Carniel et al. (2014)	DEM + 3 fiber	11	uniaxial extension (pig colon)
Carniel et al. (2015)	DEM + 2 visco	6	inflation (pig colon)
Sokolis (2017)	Fung	4	inflation-extension (rat small intestine)
	Chuong and Fung	7	
	Tong and Fung	7	
Patel et al. (2018)	N-H + 4 fiber	9	inflation-extension (pig colon)
Puértolas et al. (2020)	N-H + 2 fiber	4	biaxial extension (pig colon)
	N-H + 2 dis fib	5	
	N-H + 4 fiber	8	
	N-H + ODF (Von Mises)	5	
	N-H + ODF (Bingham)	5	
Zhao et al. (2020)	N-H + ODF (diff. tensor)	4 *	biaxial extension + inflation (mouse colon)
